# Supplementary material for: Time-series transcriptome analysis identified differentially expressed genes in broiler chicken infected with mixed Eimeria species
Source: Front Genet. 2022 Aug 8;13:886781. doi: 10.3389/fgene.2022.886781 (PMC9393255; doi:10.3389/fgene.2022.886781)
Supplement: Supplementary file 2 [file DataSheet1.ZIP › 4dpi_KEGG.Gsea.1625100298584/KEGG_CELL_ADHESION_MOLECULES_CAMS.html]

Details for gene set KEGG\_CELL\_ADHESION\_MOLECULES\_CAMS[GSEA]

|  || Dataset | TMM\_4dpi\_gct\_format\_4dpi\_gct\_format.Class\_4dpi.cls #PC\_versus\_NC.Class\_4dpi.cls #PC\_versus\_NC\_repos |
| Phenotype | Class\_4dpi.cls#PC\_versus\_NC\_repos |
| Upregulated in class | 0 |
| GeneSet | KEGG\_CELL\_ADHESION\_MOLECULES\_CAMS |
| Enrichment Score (ES) | -0.3509948 |
| Normalized Enrichment Score (NES) | -1.3918605 |
| Nominal p-value | 0.03508772 |
| FDR q-value | 0.3114855 |
| FWER p-Value | 0.9962 |
Table: GSEA Results Summary

  

Fig 1: Enrichment plot: KEGG\_CELL\_ADHESION\_MOLECULES\_CAMS      
 Profile of the Running ES Score & Positions of GeneSet Members on the Rank Ordered List

  

| SYMBOL | TITLE | RANK IN GENE LIST | RANK METRIC SCORE | RUNNING ES | CORE ENRICHMENT || 1 | CD274 | na | 682 | 0.830 | -0.0249 | No |
| 2 | CLDN7 | na | 832 | 0.746 | -0.0085 | No |
| 3 | PDCD1LG2 | na | 978 | 0.679 | 0.0057 | No |
| 4 | CD99 | na | 1157 | 0.615 | 0.0147 | No |
| 5 | ITGA6 | na | 1541 | 0.510 | 0.0024 | No |
| 6 | ALCAM | na | 1599 | 0.495 | 0.0168 | No |
| 7 | NLGN3 | na | 2341 | 0.370 | -0.0309 | No |
| 8 | NFASC | na | 2364 | 0.367 | -0.0185 | No |
| 9 | CNTNAP1 | na | 2415 | 0.359 | -0.0087 | No |
| 10 | CD34 | na | 2505 | 0.348 | -0.0027 | No |
| 11 | CD226 | na | 2666 | 0.327 | -0.0034 | No |
| 12 | CD8A | na | 2721 | 0.320 | 0.0044 | No |
| 13 | ITGB1 | na | 3030 | 0.277 | -0.0106 | No |
| 14 | SDC2 | na | 3286 | 0.245 | -0.0225 | No |
| 15 | ITGB8 | na | 3541 | 0.214 | -0.0354 | No |
| 16 | VCAN | na | 3581 | 0.209 | -0.0306 | No |
| 17 | CNTN1 | na | 3667 | 0.201 | -0.0299 | No |
| 18 | CLDN11 | na | 3926 | 0.175 | -0.0448 | No |
| 19 | CLDN23 | na | 4021 | 0.166 | -0.0462 | No |
| 20 | CDH1 | na | 4042 | 0.164 | -0.0415 | No |
| 21 | PTPRM | na | 4220 | 0.149 | -0.0505 | No |
| 22 | PECAM1 | na | 4383 | 0.135 | -0.0589 | No |
| 23 | CDH5 | na | 4542 | 0.121 | -0.0674 | No |
| 24 | CD80 | na | 4648 | 0.111 | -0.0720 | No |
| 25 | CDH4 | na | 4793 | 0.098 | -0.0802 | No |
| 26 | CLDN2 | na | 5140 | 0.066 | -0.1066 | No |
| 27 | CLDN1 | na | 5284 | 0.055 | -0.1165 | No |
| 28 | NRCAM | na | 5480 | 0.038 | -0.1313 | No |
| 29 | CLDN5 | na | 5865 | 0.004 | -0.1633 | No |
| 30 | NECTIN1 | na | 5879 | 0.004 | -0.1643 | No |
| 31 | VCAM1 | na | 6006 | -0.007 | -0.1746 | No |
| 32 | CD2 | na | 6246 | -0.026 | -0.1935 | No |
| 33 | OCLN | na | 6770 | -0.068 | -0.2347 | No |
| 34 | CTLA4 | na | 7112 | -0.100 | -0.2594 | No |
| 35 | NECTIN3 | na | 7357 | -0.123 | -0.2751 | No |
| 36 | ITGAV | na | 7369 | -0.124 | -0.2712 | No |
| 37 | CDH15 | na | 7517 | -0.136 | -0.2782 | No |
| 38 | ITGB2 | na | 7941 | -0.173 | -0.3070 | No |
| 39 | GLG1 | na | 8105 | -0.189 | -0.3133 | No |
| 40 | CADM3 | na | 8219 | -0.200 | -0.3150 | No |
| 41 | CD40LG | na | 8222 | -0.200 | -0.3074 | No |
| 42 | CD86 | na | 8523 | -0.232 | -0.3235 | No |
| 43 | JAM3 | na | 8852 | -0.268 | -0.3406 | Yes |
| 44 | CLDN3 | na | 8863 | -0.269 | -0.3310 | Yes |
| 45 | ITGA8 | na | 8864 | -0.269 | -0.3205 | Yes |
| 46 | PTPRC | na | 8879 | -0.271 | -0.3112 | Yes |
| 47 | SDC4 | na | 9133 | -0.300 | -0.3208 | Yes |
| 48 | NRXN1 | na | 9190 | -0.307 | -0.3136 | Yes |
| 49 | ITGA9 | na | 9257 | -0.316 | -0.3068 | Yes |
| 50 | CD276 | na | 9585 | -0.361 | -0.3202 | Yes |
| 51 | ICOS | na | 9720 | -0.376 | -0.3169 | Yes |
| 52 | CADM1 | na | 9880 | -0.395 | -0.3148 | Yes |
| 53 | NCAM1 | na | 10023 | -0.418 | -0.3105 | Yes |
| 54 | CDH2 | na | 10036 | -0.419 | -0.2953 | Yes |
| 55 | SDC3 | na | 10185 | -0.443 | -0.2905 | Yes |
| 56 | SDC1 | na | 10194 | -0.444 | -0.2740 | Yes |
| 57 | CDH3 | na | 10206 | -0.445 | -0.2576 | Yes |
| 58 | CD28 | na | 10233 | -0.450 | -0.2423 | Yes |
| 59 | NEO1 | na | 10324 | -0.465 | -0.2318 | Yes |
| 60 | JAM2 | na | 10362 | -0.472 | -0.2166 | Yes |
| 61 | NRXN3 | na | 10411 | -0.481 | -0.2020 | Yes |
| 62 | CD6 | na | 10422 | -0.482 | -0.1841 | Yes |
| 63 | PTPRF | na | 10715 | -0.541 | -0.1876 | Yes |
| 64 | NEGR1 | na | 10754 | -0.549 | -0.1695 | Yes |
| 65 | CD40 | na | 10769 | -0.552 | -0.1492 | Yes |
| 66 | ITGA4 | na | 10879 | -0.577 | -0.1360 | Yes |
| 67 | CD4 | na | 11000 | -0.608 | -0.1225 | Yes |
| 68 | ICOSLG | na | 11035 | -0.617 | -0.1014 | Yes |
| 69 | SELE | na | 11370 | -0.734 | -0.1009 | Yes |
| 70 | CLDN10 | na | 11621 | -0.890 | -0.0873 | Yes |
| 71 | MADCAM1 | na | 11667 | -0.933 | -0.0549 | Yes |
| 72 | PDCD1 | na | 11692 | -0.955 | -0.0199 | Yes |
| 73 | CNTN2 | na | 11858 | -1.197 | 0.0127 | Yes |
Table: GSEA details [plain text format]

  

Fig 2: KEGG\_CELL\_ADHESION\_MOLECULES\_CAMS      
 Blue-Pink O' Gram in the Space of the Analyzed GeneSet

  

Fig 3: KEGG\_CELL\_ADHESION\_MOLECULES\_CAMS: Random ES distribution      
 Gene set null distribution of ES for **KEGG\_CELL\_ADHESION\_MOLECULES\_CAMS**
